# A118 UPPER GASTROINTESTINAL HEMORRHAGE SECONDARY TO SUPERIOR MESENTERIC ARTERY PSEUDOANEURYSM: CASE REPORT AND REVIEW OF LITERATURE

**DOI:** 10.1093/jcag/gwab049.117

**Published:** 2022-02-21

**Authors:** H Guo, J Stach, R Panaccione, P J Belletrutti

**Affiliations:** 1 Medicine, University of Calgary, Calgary, AB, Canada; 2 Gastroenterology, University of Calgary, Medicine Hat, AB, Canada; 3 University of Calgary, Calgary, AB, Canada

## Abstract

**Background:**

Visceral artery pseudoaneurysms (VAPAs) are rare with an estimated incidence of 0.1%-0.2%. Due to various etiologies, a tear occurs in the vessel wall with subsequent formation of a peri-artery hematoma. A ruptured VAPA is a clinical emergency due to life-threatening hemorrhage and is associated with mortality rates of 25%-75%.

**Aims:**

We report a case of upper gastrointestinal (GI) hemorrhage secondary to a ruptured superior mesenteric artery (SMA) pseudoaneurysm. A review of the literature regarding management of VAPAs and SMA pseudoaneurysms was performed using relevant medical subject headings on PubMed.

**Methods:**

A 66-year-old woman presented to hospital with sudden large volume hematemesis and melena. Her daily medications included Aspirin and Atorvastatin. She had no prior history of peptic ulcers or chronic liver disease. She was found to be tachycardic and hypotensive. Initial investigations demonstrated a hemoglobin of 42g/L and a blood urea nitrogen of 17.5mmol/L. She was resuscitated and referred for an emergent upper endoscopy. On endoscopy, in the third portion of the duodenum, a 4cm solid-appearing subepithelial lesion with central umbilication and an apparent visible vessel was identified. Upon inspection of the lesion, the umbilicated area spontaneously began spurting blood (Image 1). A hemoclip was immediately placed next to the lesion for localization, then hemostatic powder was applied to the area. An immediate computerized tomography (CT) angiography of the abdomen revealed a 3.9 x 2.1 cm pseudoaneurysm arising from the superior mesenteric artery impressing upon the duodenum.

**Results:**

Transcathether arterial embolization of the SMA pseudoaneurysm was performed, during which two Nester coils were deposited in the ileocolic outflow vessel. A covered endovascular stent was also deployed across the culprit arterial branch to exclude the pseudoaneurysm. Following the procedure, the patient stabilized and had no further GI bleeding. Traditionally, visceral angiography has been the gold standard diagnostic test for VAPAs, but has now been supplanted by CT angiography. Treatment strategies of VAPAs can be broadly separated into endovascular methods (coils, vascular plugs, stents, liquid embolic agents) and surgical methods (aneurysmectomy with patching, end-to-end anastomosis, bypass grafting).

**Conclusions:**

SMA pseudoaneurysms are a rare yet life-threatening cause of GI bleeding. Endoscopically, they resemble solid subepithelial masses, such as GI stromal tumor, nerve sheath tumor or a lipoma, which may lead to inappropriate attempts to biopsy the lesion or apply direct endoscopic therapy. Prompt diagnosis with imaging, such as CT angiography, is paramount with a view to definitive treatment of the pseudoaneurysm via endovascular methods.

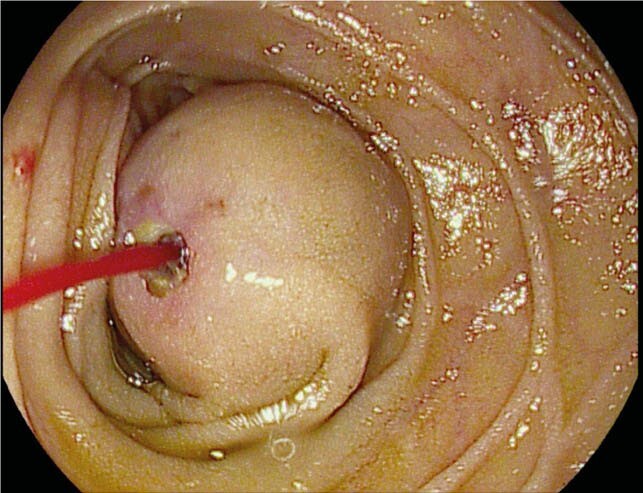

Actively hemorrhagic SMA pseudoaneurysm

**Funding Agencies:**

None

